# Information-Theoretic Method for Assessing the Quality of Translations

**DOI:** 10.3390/e24121739

**Published:** 2022-11-29

**Authors:** Boris Ryabko, Nadezhda Savina

**Affiliations:** 1 Federal Research Center for Information and Computational Technologies, 630090 Novosibirsk, Russia; 2 Department of Information Technologies, Novosibirsk State University, 630090 Novosibirsk, Russia

**Keywords:** translation of literary texts, data compression, hypothesis testing, information-theoretic classification method

## Abstract

In recent years, the task of translating from one language to another has attracted wide attention from researchers due to numerous practical uses, ranging from the translation of various texts and speeches, including the so-called “machine” translation, to the dubbing of films and numerous other video materials. To study this problem, we propose to use the information-theoretic method for assessing the quality of translations. We based our approach on the classification of sources of text variability proposed by A.N. Kolmogorov: information content, form, and unconscious author’s style. It is clear that the unconscious “author’s” style is influenced by the translator. So researchers need special methods to determine how accurately the author’s style is conveyed, because it, in a sense, determines the quality of the translation. In this paper, we propose a method that allows us to estimate the quality of translation from different translators. The method is used to study translations of classical English-language works into Russian and, conversely, Russian classics into English. We successfully used this method to determine the attribution of literary texts.

## 1. Introduction

Translations from one language to another play an important role in the modern information society. Translations of a wide variety of data, including literary works, texts from newspapers and TV programs, and the content of various social networks, are used for dubbing films to maintain dialogues when participants speak different languages. Naturally, such a widespread use of translations arouses interest and contributes to the development of new translation methods, for example, various machine translation programs, and, in turn, the comparison of their quality [1,2,3]. It should be noted here that for centuries the task of comparing the quality of translations of literary works was solved by literary scholars using their traditional methods, and by now, significant material has been accumulated both on the analysis of specific individual translations of classical authors and on the development of general principles for the analysis of translations [4,5,6,7,8]. It is important to note the appearance in recent years of formal, mathematical methods for analyzing texts [9,10,11,12,13,14]. Nevertheless, despite a significant number of works devoted to the analysis of literary works, including translations, there are few generally accepted concepts and formal definitions in this area, for example, what a quality translation is. Among the fairly recognized and well-known theories, we present the classification of text variability sources proposed by Kolmogorov [15]: information content, form, and unconscious author’s style. Moreover, the concept of unconscious author’s style can also be applied to a separate literary work. In the case of translated works, the question of unconscious author’s style is far from clear. The fact is that, apparently, the “style” of the text of the translation is created by both the author and the translator. It is interesting to note that the question of the influence of the translator on the style of the translated work has been studied by literary critics. However, they did not offer any approaches to quantify the author’s and translator’s contribution to the style of translation. In this paper, we propose a new method for the comparative analysis of two characteristics of translation quality, based on a comparative quantitative analysis of the unconscious style of translation texts. First, we quantify the contribution of translators to the unconscious author’s style by comparing different translations of the same work. Secondly, we indirectly compare the contribution of the author of the work and the translator by analyzing translations of the same work by different authors. The proposed method is based on the approach developed by the authors [14] for the quantitative assessment of the writer’s unconscious author’s style. This method has been successfully applied to the attribution of literary works [14]. This article provides a description of the method and its discussion based on the analysis of the translation of English-language literary works into Russian, and, conversely, Russian classics into English. The choice is also due to the fact that the results obtained by the proposed method can be compared with the opinions of literary critics. In general, we can say that the results obtained by the proposed formal method are confirmed by the opinion of literary critics.

## 2. Description of the Method of Quantitative Assessment of the Author’s Unconscious Style

### 2.1. Main Idea of the Method

Modern information technology often uses archivers, or data compression methods. Archivers are based on so-called universal codes, which in turn are based on information theory, formal grammars, and some other concepts Archivers take text data as input and “compress” it, i.e., convert it into shorter text so that it can be decompressed into the original text. To compress it, archivers find unequal frequencies of characters and character combinations and use other text patterns.

This paper describes a scheme for applying the information-theoretic approach. Let there be three texts $T_{1},T_{2},$ and $T_{3}$. We know that $T_{1}$ and $T_{2}$ were generated by different sources of information, respectively, $S_{1}$, $S_{2}$, and $T_{3}$ were generated by either $S_{1}$ or $S_{2}$. We combine the texts into pairs $T_{1}T_{3}$ and $T_{2}T_{3}$ and compress both pairs. We also separately compress the files $T_{1}$ and $T_{2}$, after which we calculate the differences in the lengths of the compressed files $T_{1}T_{3}$ and $T_{1}$ and, similarly, $T_{2}T_{3}$ and $T_{2}$. If the difference between the lengths of the compressed files $T_{1}T_{3}$ and $T_{1}$ is less than $T_{2}T_{3}$ and $T_{2}$, then we conclude that the text $T_{3}$ was generated by the information source $S_{1}$. If the difference between the lengths of the compressed files $T_{2}T_{3}$ and $T_{2}$ is less, then the pair $T_{2}$ and $T_{3}$ was generated by $S_{2}$. This result occurs because the archiver, when compressing the appended part, that is $T_{3}$, uses the statistical features found by it when compressing the first part, namely $T_{1}$ or $T_{2}$. Therefore, $T_{3}$ is compressed better with text that generated by the same source of information.

This approach was proposed by Tehan [16] and further developed in [17,18,19]. In [20,21], this idea was applied to construct a statistical method for classifying texts, which makes it possible to determine the reliability of the obtained conclusions. This scheme was quite successfully used by the authors to solve problems of text attribution in [14], where it was experimentally shown that each author has their own individual style, which is quite accurately manifested in their text of 4 kB (about two pages). Based on this fact, we apply the same scheme for solving the problems of analyzing the quality of translations as for attribution.

### 2.2. Description of the Method on the Example of Establishing Unconscious Author’s Style for English-Speaking Writers

Let us move on to the description of the use of the scheme presented above: there are *N* different texts $T_{1},T_{2},\ldots ,T_{N}$ (for example, works of different writers). Each text $T_{i}$ is presented in the form of two samples, called training ($X_{i},i=1,\ldots ,N$) and experimental, which, in turn, consists of *m* parts (slices), which we denote by $Y_{ij},i=1,\ldots ,N;\;j=1,\ldots ,m$. We compiled a sample of texts from the works of Poe, London, Lawrence, Kipling, Dickens, and Stevenson; N=6,m=16. From the works of these authors, we made 6 training samples $X_{1},X_{2},\ldots ,X_{6}$ of 64 kB each. Then, we made test samples—16 files $Y_{1j,}\;j=1,\ldots ,16,$ 4 kB each, from Poe’s works, $Y_{2}j,j=1,\ldots ,16$, from London’s works, …, and $Y_{6j},\;j=1,\ldots ,16,$ from Stevenson’s writings. Then, we alternately “compressed” the file $Y_{11}$ with the training samples $X_{1},X_{2},\ldots ,X_{6}$. Next, we determined with which of the samples the file is “better” compressed (i.e., we calculated $d\left(X_{1}Y_{11}\right)-d\left(X_{1}\right),\ldots ,d\left(X_{6}Y_{11}\right)-d\left(X_{6}\right)$ and found *i* for which $d\left(X_{i}Y_{11}\right)-d\left(X_{i}\right)$ is minimal. Similarly, we processed all $Y_{ij},i=1,\ldots ,6,j=1,\ldots ,16$.

Let us explain the meaning of these numbers: 16 in the upper left corner means that out of 16 files $Y_{1j},j=1,\ldots ,16$, all files “compressed” better with $X_{1}$. In other words, all 16 “slices” from Poe’s works “compressed” better with a training sample from his own work. Thus, it became clear that the author’s style of Edgar Poe is uniquely determined by a slice of 4 kB in a training sample of 64 kB. The numbers in the second row of the table mean that out of 16 files $Y_{2j},j=1,\ldots ,16,$ 12 “compressed” better with $X_{2}$. In other words, 12 “slices” from London’s works are better “compressed” with his own training sample, however, two slices are more similar to Dickens’ texts, one slice is more similar to Kipling’s text and one slice is similar to Stevenson’s text. There is a “recognizability” of the text of 12 slices out of 16.

Then, for the Table 1 (and all tables below), we calculated the chi-square statistics and the Cramer coefficient *V* as follows: for a table

$$\begin{array}{cccc} a_{1\;1} & a_{1\;2} & \ldots & a_{1\;n}\\ a_{2\;1} & a_{2\;2} & \ldots & a_{1\;n}\\ \ldots \\ a_{m\;1} & a_{m\;2} & \ldots & a_{m\;n} \end{array}$$

calculate

$$N=\sum_{i=1}^{n}\sum_{\;i=1}^{m}a_{i,j},p_{i,j}=a_{i,j}/N,\;p_{i.}=\sum_{j=1}^{n}p_{i,j},\;p_{.j}=\sum_{i=1}^{m}p_{i,j},$$

(1) $$x^{2}=\sum_{i=1}^{n}\sum_{i=1}^{m}\frac{{\left(a_{i,j}-Np_{i.}p_{.j}\right)}^{2}}{Np_{i.}p_{.j}}\;\;,\;V={\left(\left(x^{2}/N\right)/\min\left\{(m-1),(n-1)\right\}\right)}^{0.5}\;,$$

see [22]. $x^{2}$ can be used to test the null hypothesis of homogeneity, since this value asymptotically obeys the chi-square distribution with (m−1)(n−1) degrees of freedom [22]. For example, the homogeneity hypothesis for Table 1 is rejected with a significance level of 0.0001.

The Cramer coefficient *V* varies from 0 (corresponding to no association between the variables) to 1 (complete association).

We call the whole process of transition from the source texts $T_{1},T_{2},\ldots ,T_{N}$ to a table (of size NxN) the construction of a contingency table, and the contingency table itself is denoted $W(T_{1},T_{2},\ldots ,T_{N}$) or *W* (depending on the context).

As we have seen, in the cells of the contingency table, the numbers indicate the number of text slices. The authorship of each slice was attributed to one or another writer. If the method works “correctly”, i.e., the method correctly determines the style of the author by slices, then the values in the table will be concentrated mainly on the main diagonal (in the ideal case of the method, the matrix will be diagonal). Otherwise, when the slices do not give an idea of the style of the author, the values in the tables will be evenly distributed. We quantified this effect by the Cramer coefficient *V*: with a diagonal placement, the Cramer coefficient is 1, and with a uniform distribution, *V* is close to 0.

### 2.3. Selecting Method Parameters

It is important to note that we determined all the parameters during the preliminary experiments in order to choose those that maximize the Cramer coefficient. First, various archivers were considered.

Table 2 shows that LZMA gives the maximum value of the Cramer coefficient, so we used this archiver in the study.

When identifying the authorship of literary texts, some researchers use the so-called text preprocessing. When solving our problem, we also used the text preprocessing method to find the maximum Cramer coefficient based on experiments. The experimental results are given in the following Table 3:

It can be seen that the highest criterion value was achieved for texts from which only punctuation was removed. Therefore, this preprocessing was used in all other experiments. At the next stage, we experimentally determined the volumes of the training sample, one slice, and their number, focusing on the value of the Cramer coefficient, chi-square, and the total required amount of data. We explored slice sizes of 2 kB, 4 kB, and 8 kB with several training sample sizes. According to the results of the study, the size of the training sample was 64 Kb, with 16 slices of 4 Kb each. Interestingly, these values coincide with previously determined parameters for Russian-speaking writers [14].

## 3. Interpenetration of the Style of the Translator and the Style of the Author in Translation

In this short section, we show experimentally that the style of translation of a literary work really depends on both the author’s style and the translator’s, but the translation style does not coincide with either one or the other. To solve this problem, we consider the works of K. Chukovsky and M. Engelhardt, who are both famous writers and famous translators. In the following table, we show the results of a study of their unconscious author’s style as writers.

(The homogeneity hypothesis for Table 1 is rejected with a significance level of 0.0001) We see in the table that the unconscious author’s style is reliably determined from their own novels. Let us now consider the translations of Chukovsky and Engelhardt of M. Twain’s novel *The Adventures of Tom Sawyer.* We preprocessed these translations (see Table 3 and Table 4) in the same way as we processed their own works, shown earlier in Table 4.

Comparing these tables, we see that the situations are completely different. Table 4 shows that the style of the author is determined almost unmistakably per slice of 4 kB (training sample 64 kB). In all cases, the author is correctly identified, and the Cramer coefficient is equal to 1. On the contrary, Table 5 indicates an extremely unreliable definition of the translator’s style. The Cramer coefficient is significantly less than in the previous test. It is interesting to note that Table 5 also provides some additional information—the table shows that in Chukovsky’s translations his own author’s writing style manifests itself more than in Engelhardt’s translations.

Let us describe another experiment with the same writers, showing that the text of the translation has its own style, different from the style of the translator. In this experiment, we formed training samples—$X_{1}$ and $X_{2}$—from the literary works of Chukovsky and Engelhardt and testing samples $Y_{1.1},Y_{1.2},\ldots ,Y_{1.16}$, $Y_{2.1},Y_{2.2},\ldots ,Y_{2.16}$ from translations of *The Adventures of Tom Sawyer* by M. Twain. The results are shown in the following Table 6.

If we compare with Table 4, we see that the results are radically different: Engelhardt’s translations are more similar to Chukovsky’s texts than to his own works. In other words, with the same parameters, the definition of the translation style, if possible, is with a very large number of errors. In our opinion, this fact indicates that the style of the writer differs significantly from the style of his own translations.

## 4. The Degree of Preservation of the Author’s Style by the Translator

In this section, we consider the issue of preserving the styles of original authors when translating their works by a particular translator. We describe the proposed method step by step, illustrating with examples from different situations.

**1.**  **Step—Input data.** We collect translations from one language into another of various texts made by one translator.
**2.**  **Step—Algorithm operation.** Based on these translations, we make training and testing samples, build contingency tables *W*, and calculate the Cramer coefficient *V*.
**3.**  **Step—Interpretation.** In general, the smaller the *V*, the weaker the differences in the style of translations. The content of *W* is interesting because the “own” style of some writers in translations can be revealed to be much weaker than other writers. For example, as we see below, the style of Gogol’s works in English translations is much less preserved than, for instance, the style of Dostoevsky’s works.
**4.**  **Step—Comparison of translators.** Similarly, we can process the translations of several translators and compare them using the received *W* and *V*.

Let us start with a study first of the translations of British and American classics into Russian made by the above-mentioned well-known Russian translators K. Chukovsky and M. Engelhardt and then the translations of Russian classics by famous English-speaking translators: Garnett, Piviar, Volokhonskaya, and Hogardt. Here and below, the volume of the training sample is 64 kB, and there are 16 slices, each 4 kB.

(The homogeneity hypothesis for Table 7 (and following Table 8, Table 9 and Table 10) is rejected with a significance level of 0.0001).

The table allows us to conclude that the translations of K. Chukovsky’s works by O’Henry, Twain, and Wilde differ in style quite reliably, that is, the translator retains the unconscious author’s style of the works.

Let us now consider the translations of the works of the Russian classics Dostoevsky, Turgenev, and Gogol by the famous translator from Russian into English, Charles James Hogarth. The contingency table is presented below.

From the table, we see that the style of Dostoevsky’s translations differs perfectly from the style of translated texts of other writers: 16 out of 16 of his slices are closest to the texts of his translations. Turgenev’s style is transmitted somewhat worse—two slices from translations of his works are more similar to Gogol’s works. In addition, Gogol’s style is conveyed the worst of all—six slices from his works are more similar to other writers. On the whole, the author’s style is worse preserved in Hogarth’s translation than in Chukovsky’s translations (Table 7). This fact is confirmed by the values of the Cramer coefficient—0.79 and 0.94, respectively. It should be noted that, apparently, not only translators preserve the style of the author in translation in different ways, but there are “difficult” authors whose style is difficult for all translators to maintain in translation. Among Russian writers of the 19th century, Gogol’s authorial style was and remains the most difficult to translate, which is confirmed by the opinion of many literary critics [23]. Thus, the American critic K. Proffer [24] very briefly and peculiarly called Gogol’s style “a nightmare for a translator”, arguing that a feature of Gogol’s style is a sense of humor and irony, a slight hint of sarcasm, which is difficult to translate into other languages. Gogol is a master of puns. Gogol’s works are considered by all translators to be the most difficult to translate, and it is almost impossible to achieve an adequate translation. Difficulties in translating Gogol’s texts are shown in the following contingency tables, which show data on the translations of C. Garnett and Pivear and Volokhonskaya.

We can see from the above tables that, on average, the style of translations of works is preserved quite well (as evidenced by the high values of the Cramer coefficient), but in Gogol’s translations into English, slices of texts from his works are often “closer” to translations of other authors. This fact indicates that his author’s style is transmitted by the translator much worse (in other words, the author’s style is worse preserved in translation).

We found a similar effect in translations from English into Russian. In Table 11, we can see that Engelhart’s translations convey Dickens’ authorial style to a lesser extent than Doyle’s style and Twain’s style.

## 5. Comparative Analysis of the Influence of the Translator’s Style on the Translated Text

As we noted earlier, each translator has their own unconscious author’s style, based on individual personal vocabulary and syntax, sentence construction, the use of figures of speech, and idiomatic phrases. There is a widespread opinion among literary scholars, and indeed among a wide range of readers, that if the literary style of the translator is less recognizable or noticeable, then the quality of the translation is better (and, conversely, the translation is bad if the style of the translator “overshadows” the style of the author). In this section, we propose a method for quantitatively comparing the “contribution” of translators to the style of translation. In a sense, this allows you to compare the quality of translations of one work by different authors. As before, we first describe the proposed method step by step, and then we give examples that demonstrate different situations.

**Input data:** Translations from one language to another of the same text made by different translators.

**First step:** We process translations of one work made by different translators to obtain a contingency table *W* and calculate the Cramer coefficient *V*.

**Second step:** We analyze and interpret the received data. At the same time, it should be noted that if *V* is less, then the differences in the style of translation of a given work are weaker and the styles of translators are less noticeable. The content of *W* is also of interest because the contribution of some translators to the value of *V* can be expressed much mroe weakly than others.

Let us first consider an example of the translation of M. Twain’s novel into Russian by Chukovsky and Yasinsky in Table 12.

As we have already noted, if the contribution of translators to the style of translation was insignificant (ideally, the contribution of the translator was completely absent), then the values in all cells of the table would be 8. In this table, the situation is close to ideal, as evidenced by the values of the Cramer coefficient *V*. Therefore, the translator’s contribution to the translated text is rather small.

We discovered an interesting fact: the situation with translations of Russian writers into English is completely different. First, consider the calculation results shown in the following Table 13, Table 14 and Table 15.

From the tables, we see that the precise identification of the translator’s style in translations of Russian classics into English clearly contrasts with the situation with translations of English literature into Russian. This somewhat unexpected fact is confirmed by the opinion of many writers and literary critics who are fluent in both languages. Thus, the well-known writer, Nobel laureate I. Brodsky, said that the dominance of the translator’s style in translations of Russian classics leads to the unification of the styles of all Russian writers [25].

## 6. Conclusions

In this paper, we propose a method for quantitative assessment of the quality of translation, which solves two problems: (1) quantitative assessment of the degree of preservation of the author’s style in translations and (2) quantitative assessment of the translator’s contribution to the translated text. The method is “tuned” to the analysis of translations of classical English-language literature into Russian and, conversely, of Russian classic writers into English. Comparison of the obtained results with the opinion of literary critics, who have numerous works in these areas, allows us to conclude that the proposed method is adequate and effective. In our opinion, the proposed method, with appropriate parameter settings, can be applied to the analysis of “machine translation”, the quality of film duplication, and in other areas where the task of evaluating the quality of translation arises.

## Figures and Tables

**Table 1:** Results of the experiments. The data obtained for the LZMA archiver, training set ($X_{ij}$) 64 kB, and slice ($Y_{ij}$) 4 kB. At the same time, we carried out the so-called preprocessing of texts: we removed proper names and punctuation marks from all texts.

	1	2	3	4	5	6
Eureka (Poe)	16	0	0	0	0	0
Son of the Wolf (London)	0	12	0	1	2	1
Women in Love (Lawrence)	0	1	10	2	2	1
The Jungle Book (Kipling)	0	3	0	12	0	1
The Adventures of Oliver Twist (Dickens)	0	0	0	0	16	0
Treasure Island (Stevenson)	0	0	0	0	3	13

**Table 2:** The Cramer coefficient values for different archivers. (Data from Table 1).

Archiver	Cramer Coefficient *V*
BZIP2	0.6472
LZMA	0.9041
DEFLATE	0.8131

**Table 3:** Values of the Cramer coefficient for different types of text preprocessing. (Data from Table 1).

Type of Text Preprocessing	*V* Coefficient
With punctuation, with proper nouns, and with capital letters	0.8585
Without punctuation, with proper nouns, and with capital letters	0.9203
Without proper nouns, without punctuation, and with capital letters	0.9108
Without capital letters, without punctuation, and without proper nouns	0.8965

**Table 4:** The results of recognition of the author’s style of the writers Chukovsky and Engelhardt. The Cramer coefficient V=1.

	K. Chukovsky (Writer)	M. Engelhardt (Writer)
Sunny	16	0
Louis Pasteur	0	16
His life and scientific activity		

**Table 5:** Results of translator style identification of Chukovsky and Engelhardt. The Cramer coefficient V=0.43.

	K. Chukovsky (Translator)	M. Engelhardt (Translator)
*The Adventures of Tom Sawyer*	13	3
(M. Twain, translated by K. Chukovsky)		
*The Adventures of Tom Sawyer*	8	8
(M. Twain, translated by M. Engelhardt)		

**Table 6:** Results of translator style identification. The Cramer coefficient V=0.62.

	K. Chukovsky (Writer)	M. Engelhardt (Writer)
*The Adventures of Tom Sawyer*	15	1
(M. Twain, translated by K. Chukovsky)		
*The Adventures of Tom Sawyer*	10	6
(M. Twain, translated by M. Engelhardt)		

**Table 7:** Translations by K. Chukovsky. The Cramer coefficient V=0.94.

	O’Henry	Twain	Wilde
O’Henry	16	0	0
Twain	0	16	0
Wilde	1	1	14

**Table 8:** Translations by Charles James Hogarth. The Cramer coefficient V=0.79.

	Turgenev	Dostoevsky	Gogol
Turgenev	14	0	2
Dostoevsky	0	16	0
Gogol	5	1	10

**Table 9:** Translations by C. Garnett. The Cramer coefficient V=0.88.

	Tolstoy	Dostoevsky	Gogol
Tolstoy	15	1	0
Dostoevsky	0	16	0
Gogol	2	2	12

**Table 10:** Translations by Richard Pevear and Larisa Volokhonsky. The Cramer coefficient V=0.85.

	Pasternak	Dostoevsky	Gogoll
Pasternak	15	1	0
Dostoevsky	1	15	0
Gogol	1	2	13

**Table 11:** Translations by M. Engelhardt. The Cramer coefficient V=0.88.

	Dickens	Doyle	Twain
Dickens	11	1	3
Doyle	0	16	0
Twain	1	0	15

**Table 12:** Comparative analysis of translations. The novel *The Prince and the Pauper* (Twain). The Cramer coefficient V=0.47.

	Translator K. Chukovsky	Translator I. Yasinsky
translator K. Chukovsky	9	7
translator I. Yasinsky	6	10

**Table 13:** Novel *Crime And Punishment* (Dostoevsky). The Cramer coefficient V=1.

	Translators	Translator
	Pevear and Volokhonsky	Garnett
translators Pevear and Volokhonsky	16	0
translator Garnett	0	16

**Table 14:** Novel *Dead Souls* (Gogol). The Cramer coefficient V=0.88.

	Translator	Translators
	Hogarth	Pevear and Volokhonsky
translator Hogarth	14	2
translators Pevear and Volokhonsky	1	15

**Table 15:** Novel *The Gambler* (Dostoevsky). The Cramer coefficient V=0.94.

	Translator	Translators
	Pevear and Volokhonsky	Hogarth
translators Pevear and Volokhonsky	16	0
translator Hogarth	1	15
